# Supplementary material for: A Scoping Review on Body Fluid Biomarkers for Prognosis and Disease Activity in Patients with Multiple Sclerosis
Source: J Pers Med. 2022 Aug 31;12(9):1430. doi: 10.3390/jpm12091430 (PMC9501898; doi:10.3390/jpm12091430)
Supplement: Supplementary file 1 [file jpm-12-01430-s001.zip › jpm-1860041-supplementary.pdf]

TABLE S1: cytoskeletal biomarkers

biomarker class	biomarker subtype	full name	biomarker description	body fluid/tissue	outcome	observations	n of patients	primary studies	review
Neurofilaments	NF-H	Neurofilament Heavy chain	Axonal protein, main component of the axonal cytoskeleton, reflecting acute and ongoing axonal damage	CSF	disease severity	Biomarker of accumulated axonal damage in progressive MS.<br>Predictive of more severe EDSS progression and brain atrophy	51 MS: 20 RR, 21 SP, 10 PP	[94]; [95]	[26]
				CSF	disease severity	In a cohort of CIS patients CSF NfH levels correlated with physical disability (r=0.304, p<0.05) and brain volume loss (r=-0.518, p<0.01) (1 year of follow-up)	67 CIS	[96]	[25]
				serum	RR to SP	-Serum NFH can be detected more likely in SPMS than in RRMS, indicating that it can be a prognostic indicator of the disease outcome. [97]<br>-The presence of serum NFH is associated with higher EDSS and T2 cerebral MRI lesion load. [97]<br>-NfH levels where higher in patients with progressive disease (PP and SP) compared to RR (p<0.05), and correlated weakly with all disability scores (EDSS, ambulance and 9-hole peg test), indicating that it is a poor prognostic sign [98]<br>-This finding was not confirmed in another study [99]	23 SP/PP, 11 RR [98]<br>21 RR, 20 SP, 10 PP [99]	[97-99];	[10, 11];
Neurofilaments	NF-L and NF-H	Neurofilament Light chain; heavy chain	Axonal proteins, main components of the axonal cytoskeleton.		disability in PP and SP	In a cohort of patients with progressive NfH was a predictor of ongoing disability (modest correlation with MSSS, r2=0.17, p<0.05)	21 PP, 10 SP	[100]	[25, 40]
Neurofilaments	NF-L	Neurofilament Light chain	Axonal proteins, main components of the axonal cytoskeleton	CSF	CIS to MS	-Higher NFL levels in CSF at baseline in patients converting to RRMS (n = 9; median NFL, 2332 ng/L; interquartile range, 1348–2810 ng/L) during 2 years of follow-up compared with patients who did not convert to RRMS (n = 10; median NFL, 245 ng/L; interquartile range, 190–747 ng/L) (P = 0.001). [101]<br>-NfL levels, show a significant difference between CIS-CDMS (1,553.1 [1,208.7–1,897.5] ng/L) and CIS-CIS (499.0 [168.8–829.2] ng/L) (p <0.0001) [102]<br>-A Cox hazard regression model showed that only CIS patients with increased CSF levels of NFL were associated with earlier conversion to CDMS (HR (95% CI): 2.69 (1.75 – 4.15); p < 0.0001) [63]<br>-The conversion to MS occurred in half of the CIS patients (19/39) during the follow-up (CIS–MS group). Median time to conversion was 10.6 months (interquartile range: 5.2–13 months) [103]<br>-The neurofilament light chain levels increased in CIS patients who converted to MS [104]	109 CIS [63]<br>19 CIS [101]<br>68 CIS [102]<br>39 CIS [103]<br>38 CIS [104]	[63, 101-104]	[40]
Neurofilaments	NF-L	Neurofilament Light chain	Axonal proteins, main components of the axonal cytoskeleton	serum	CIS to MS	-When FC and NC were grouped together, NfL concentration was positively associated with presence of Gd+ lesions (OR=2.69; 95% CI 1.13 to 6.41; p=0.026), increasing T2 lesion load (OR=2.36; 95% CI 1.21 to 4.59; p=0.011), increasing EDSS (OR=2.54; 95% CI 1.21 to 5.31; p=0.013), but not OCB status (OR=1.66; 95% CI 0.75 to 3.70; p=0.214). [105]<br>-The screening phase evaluated patients developing clinically definite MS (CIS-CDMS) a 2-year minimum follow-up. In the group of patients who evolved from CIS to MS, the mean CSF NfL level was 1,555 ng/L and in the group who remained as CIS after two years, the mean CSF NfL level was 499 ng/L (p < 0.0001). [102]	38 MS [102]<br>100 fast converters;<br>98 non converters [105]<br>222 CIS [106]	[102, 105-106]	[33, 40, 48, 49]

						-Serum NfL (median 22.0, interquartile range 11.6-40.4 pg/mL) was noticeably increased in patients with a recent relapse, with a high number of T2 and gadolinium-enhancing lesions at baseline MRI, and it was prognostic for clinically defined MS (low levels of NfL were associated with a 2-fold lower risk of disease progression). [106]			
Neurofilaments	NF-L	Neurofilament Light chain	Axonal proteins, main components of the axonal cytoskeleton	CSF	Disease severity	Significant correlations between neurofilament light levels and the multiple sclerosis severity score were found for all cases (r = 0.30, p = 0.005), for relapsing-remitting multiple sclerosis cases (r = 0.47, p < 0.001) and for cases with a recent relapse (r = 0.60, p < 0.001).	99 RRMS	[107]	[40, 49]
Neurofilaments	NF-L	Neurofilament Light chain	Axonal proteins, main components of the axonal cytoskeleton	serum	Disease severity	The increase in sNfL per EDSS unit increase was lower in PPMS/SPMS than in CIS/RRMS patients (β=1.024,95% CI=0.952–1.101 vs β=1.133, 95% CI=1.081–1.187 respectively; interaction p=0.021). [108]<br><br>Serum NfL levels above 90th percentile (of healthy controls) were an independent predictor of EDSS worsening in the subsequent year (p<0.001). Contrast enhancing and new lesions were associated with increased NfL levels (17.8% and 4.9% increase per lesion respectively; P < 0.001). [109]	Cross-sectional cohort :142 MS + Longitudinal cohort: 246 MS [108]<br>259 MS [109]	[108-109]	[40, 43, 46, 48]
Neurofilaments	NF-L	Neurofilament Light chain	Axonal proteins, main components of the axonal cytoskeleton	blood	Disease severity	NfL levels at baseline correlated with T2 lesion load and number of gadolinium enhancing T1 lesions (p < 0.0001, both).<br><br>High vs low baseline NfL levels were associated with an increased number of new or enlarging T2 lesions (ratio of mean: 2.64 [1.51-4.60]; p = 0.0006), relapses (rate ratio: 2.53 [1.67-3.83]; p < 0.0001), brain volume loss (difference in means: -0.78% [-1.02 to -0.54]; p < 0.0001), and risk of confirmed disability worsening (hazard ratio: 1.94 [0.97-3.87]; p = 0.0605).	589 RRMS	[110]	[48]
Neurofilaments	NF-L	Neurofilament Light chain	Axonal proteins, main components of the axonal cytoskeleton	CSF	RIS to CIS or MS	In univariable Cox regression models NfL levels and IgG oligoclonal bands were associated with increased risk for conversion to CIS [hazard ratio (HR) = 1.01, 95% confidence interval (CI) 1.00–1.03, P=0.021 for NfL; HR = 10.31,95% CI 1.37–76.61, P=0.024 for oligoclonal bands] and multiple sclerosis (HR = 1.02, 95% CI 1.00–1.03, P=0.005 for NfL; HR = 9.29, 95% CI 1.24–69.41, P=0.030 for oligoclonal bands).<br><br>In multivariable Cox regression models, NfL levels and oligoclonal bands were independent predictors of conversion to CIS (HR = 1.02, 95% CI 1.00–1.04, P=0.019 for NfL; HR = 14.70, 95% CI 1.80–120.15, P=0.012 for oligoclonal bands) and multiple sclerosis (HR = 1.03, 95% CI 1.01–1.05, P=0.003 for NfL; HR = 8.86, 95% CI 1.04–75.61, P=0.046 for oligoclonal bands) in RIS patients	75 RIS	[111]	[34, 44, 46]

Cytoskeletal component	GFAP	Glial fibrillary acidic protein	Component of the intermediate filaments in the cytoskeleton of astrocytes	CSF	progressio n	-elevated GFAP levels were associated with rapid disease progression (time to reach EDSS 3.0 a: HR (95% CI): 1.83 (1.01 - 3.35); p = 0.04) [63]<br>-Increased concentrations is associated with higher disability (EDSS>6.5) and diminished ambulation (p<0.01 for the comparison with patients with less severity) [112]<br>-GFAP levels correlated with disability (EDSS, r2=0.51, p<0.05) and disease progression (MSSS, r2=0.47, p<0.05, 2 measures at 8-10 years of distance) [113]<br>-GFAP levels correlated with disability (EDSS, r2=0.73, p<0.001) and are higher in SPMS (p<0.001) [114]	109 CIS, 192 RRMS [63]<br>20 RR, 21 SP, 10 PP [112];<br>15 RR, 10 SP [113];<br>66 MS [114];	[63, 112-114]	[12, 13, 23, 27]
Cytoskeletal component	Tau	Tau protein	microtubule associated protein. It promotes microtubules assembly and stability.	CSF	progressio n	-Patients with higher tau levels at baseline experienced more rapid disability progression:<br>in survival analysis, patients with a Tau protein concentration in the CSF above the median (118 pg/ml) experienced a more rapid one-point increase in their EDSS score than those with a Tau protein concentration below the median; Log Rank=0.04.<br>-Patients with 'high' CSF-TAU had a higher EDSS score at the end of the follow-up (2.2±1.0 vs. 1.2±1.0, P=0.02) (3 years)	32 RRMS	[59]	[27]

TABLE S2: Vitamin D

biomarker full name	biomarker description	body fluid	outcome	observations	n of patients	primary studies	review
25-hydroxy (OH) vitamin D	Low levels of Vitamin D seem to be correlated with higher risk of MS	serum	CIS to MS	-In a group of 100 patients with CIS those with low (below the 25th percentile) and very low (below the 10th percentile) levels of 25-OH Vitamin D were at higher risk of conversion to definite MS after a median follow-up of 7.7 years.<br>-A large multicentric study (>1000 CIS cases) with a median follow-up of 4.3 years showed the same result in the univariate analysis, but this association was mitigated when controlling for the presence of OCB, age and number of T2 lesions on MRI.	1047 CIS [1]<br>100 CIS [115]	[1, 115]	[25]
			CIS to MS	In a group of >450 CIS patients followed up for 5 years higher vitamin D levels predicted lower hazard of conversion to clinical definite MS (p=0.048)<br>The hazard of conversion decreased with increasing serum vitamin D levels by over 50% for a 20 ng/ml increment in 25(OH)D	468 CIS<br>(292 treated with interferon, 176 with placebo)	[64]	[11, 21, 25]
			disease activity	In a group of >450 CIS patients followed up for 5 years higher vitamin D levels predicted less disease activity. A 20ng/ml increment in the mean serum levels in the first year predicted:<br>-57% lower rate of new active lesions (p=0.002)<br>-27% lower relapse rate (not significant)<br>-25% lower yearly increase in T2 lesions volume<br>-0.41% lower yearly loss in brain volume	468 CIS<br>(292 treated with interferon, 176 with placebo)	[64]	[11, 21, 25, 39]
			disease activity	Other studies reported a decrease in relapse rate at increased 25(OH)D levels.<br>They report variable percentage of decrease in relapse rate for each 25 nmol/L increase in 25(OH)D: (14%-34%) in patients with CIS or RRMS [1, 116-121].<br>However, in the largest cohort with RRMS onset (1482 RR), the 25(OH)D levels did not predict subsequent long-term relapse rate [120].	N patients (years of follow-up):<br>1047 CIS (4.3) [1]<br>145 RR (0.5) [116]<br>110 pediatric MS (1.7) [117]<br>469 RR/CIS (5) [118]<br>73 RR (1.7) [119]<br>1482 RR (2) [120]<br>340 young RRMS (3) [121]	[1, 116-121]	[39]
			progression	A 20ng/ml increment in the mean serum levels in the first year predicted:<br>-20% lower yearly increase in T2 lesions volume (P=0.00002)<br>-0.27% lower yearly loss in brain volume (not significant)	468 CIS<br>(292 treated with interferon, 176 with placebo)	[64]	[11, 21, 25]
			disease activity	Inverse correlation between vitamin d status and MS activity	145 RRMS [116];<br>178 MS [122];<br>469 MS [118]	[116, 118, 122]	[11]

TABLE S3: Antibodies

biomarker class	biomarker subtype	full name	biomarker description	body fluid	outcome	observations	n of patients	primary studies	review
OCB	IgM OCB	Lipid-specific immunoglobulin M oligoclonal bands	IgG and IgM antibodies are present in OCB (oligoclonal bands) in CSF. In contrast to IgG, IgM antibody OCB are present in the CSF of only 40% of MS patients	CSF	CIS to MS	Prediction of early conversion from CIS to clinically definite MS:<br>-the probability of conversion to CDMS was greater in IgM-OCB positive patients (90% of the patients had converted to CDMS after 8 months of follow-up) than in negative patients (51% of patients had converted to CDMS after 36 months of follow-up) (p = 0.0001) [123]<br>-At survival analysis, the presence of CSF-restricted IgM-OCB increased the risk of a relapse (p=0.043, HR=1.5) [124].<br>-presence of CSF IgM OCB in CIS patients is associated with subsequent MRI brain lesion accrual (p = 0.02). [125]	22 CIS followed up for 6-36 month [123];<br>205 CIS[124];<br>57 [125]	[123-125]	[15, 26, 30]
					disease progression in CIS	The presence of OCB in CIS patients predicts a more aggressive disease course:<br>-OCB positive patients had more relapses (mean, 2.0) than negative patients (mean, 0.58) (p = 0.02) and, at the end of the follow-up (3-36 months), OCB positive patients had higher EDSS scores (mean, 1.70) than negative patients (mean, 0.79) (p = 0.02) .	22 CIS followed up for 6-36 month [123];	[123]	[15]
					disease severity	In patients with progressive disease IgM OCB correlate with a higher MSSS, increased lesion number of MRI, and thinning of the retinal nerve fiber layer	58 MS [126];<br>127 MS [127]	[126, 127])	[26]
OCB	IgG OCB	immunoglobulin G oligoclonal bands	The presence of IgG OCB and/or elevated IgG index in CSF support the diagnosis of MS	CSF	CIS to MS	The presence of IgG OCB in CSF is the best validated molecular biomarkers that predicts conversion from CIS to clinical definite MS:<br>-longitudinal multicentric study with a median follow-up of 4.3 years (1047 CIS, 2 years of follow-up, p<0.001) [1]<br>-meta-analysis (71 papers, 2685 CIS, 68.6% of which with OCB+, OR for conversion to definite MS in OCB+: 9.88) [67]<br>-in a prospective cohort of 1058 CIS patients, multivariate analysis showed that OCB are an independent prognostic risk factor for conversion to CDMS (HR: 1.3, 95% CI: 1.0-1.8) [128]	1047 CIS [1];<br>2685 CIS [67];<br>1058 CIS [128]	[1, 67, 128]	[12, 21, 30]
						Prospective study (follow-up: 1998-2006). 15 patients converted to clinical definite MS.<br>-The risk of conversion is higher in CIS patients with OCB (RR=9.1).<br>-Shorter time of conversion to MS in CIS patients with both OCB and abnormal baseline MRI compared to those with only one marker (6.8 vs. 19 months).	40 CIS	[129]	[13]
						The presence of > 2 OCBs in the CSF has a positive predictive value of 97%, a negative predictive value of 84%, a sensitivity of 91% and a specificity of 94% for CIS to MS conversion [130];<br>The presence of OCBs within 3 months of CIS nearly doubled the risk of a second clinical attack over 50 months [130] or over 6 years [131].	52 CIS [130];<br>415 CIS [131]	[130, 131];	[8]

OCB	IgG OCB	immunoglobulin G oligoclonal bands		CSF	RIS to CIS	Patients with radiologically isolated syndrome presenting positive oligoclonal bands converted faster to clinically isolated syndrome and multiple sclerosis (P = 0.005 and P = 0.008, respectively)	75 RIS	[111]	[41]
autoantibodies	anti-GAGA4 IgM	anti-alpha-glucose IgM	anti-alpha-glucose IgM	serum	CIS to MS	Higher serum levels of a panel of anti-GAGA4 and/or other a-glucose glycan Ab seem to assist in the prediction of CIS patients who are prone to a relapse within 24 months:<br>Levels of anti-GAGA2, -GAGA3, -GAGA4, and -GAGA6 IgM were evaluated.<br>Kaplan– Meier survival plot comparison between cumulative risk of CIS patients who were positive for at least one IgM versus negative patients revealed significant differences P = 0.0025, up to 24 months	311 CIS + RRMS (at least 100 CIS)	[132]	[11]
autoantibodies	gMS-Classifier2	gMS-Classifier2	Algorithm based on the combination of polyclonal serum IgM antibody levels against a polymer (P63) and age	serum	CIS to MS	gMS-Classifier2 is an independent predictor for the conversion of CIS to clinically definite MS (p=0.014 at 2 years of follow-up)	75 CIS	[133]	[11]
autoantibodies	IgM anti-MOG<br>IgM anti-myelin basic protein	anti-MOG (Myelin Oligodendrocite Glicoprotein) antibodies	A class of myelin autoantigens. It has emerged as promising diagnostic biomarker in autoimmune pediatric demyelination. Its role in adults is still speculative.	serum	CIS to MS	An initial study showed that the presence of anti-MOG Ab could predict the risk of conversion from CIS to definite MS (p<0.001, 12 months follow-up) [134].<br>However further studies showed that anti MOG Ab are only detectable in case of acute disseminated encephalomyelitis [135-137] and other studies testing anti-MOG Ab predictive power revealed controversial results.	103 CIS [134]; 109 serum; 69 CSF [135]; 27 MS [136] ; 94 CIS [137]	[134-137]	[8, 11, 15, 25]
autoantibodies	Ab anti-MBP	antibody for myelin basic protein	antibody specific for myelin basic protein	serum	CIS to MS	Patients with anti-MOG and anti-MBP antibodies had relapses more often and earlier than patients without these antibodies. Only 9 of 39 antibody-seronegative patients (23 percent) had a relapse, and the mean (+/-SD) time to relapse was 45.1+/-13.7 months. In contrast, 21 of 22 patients (95 percent) with antibodies against both MOG and MBP had a relapse within a mean of 7.5+/-4.4 months, and 35 of 42 patients (83 percent) with only anti-MOG antibodies had a relapse within 14.6+/-9.6 months (P<0.001 for both comparisons with antibody-seronegative patients). [134]<br>However, this result was not confirmed by Vogt et al. [138]	103 CIS [134]; 37 CIS [138]	[134, 138]	[8]
					disease activity	anti-MBP levels correlated with the number of T2 lesions (r = 0.31, p = 0.041) and T1 lesions (r = 0.37, p = 0.016).	65 MS 37 CIS	[138]	[27]

anti-virus Ab	anti-EBNA1 Ab	anti-EBNA1 Ab	Antibodies directed against Epstein-Barr virus	serum	CIS to MS	anti-EBNA1 IgG titer may could be used as a prognostic marker for disease conversion and disability progression:-in fact Lunemann in a 7 years follow up study on CIS patients reports that IgG response to EBNA1 correlated with number of T2 lesions and the number of Barkhof criteria (specific for early conversion to MS)	147 CIS	[139]	[11]
					disease activity	Increasing level of anti-EBNA IgG associated with increased MRI disease activity (p=0.036, measured as sum of T1 Gd+ lesions plus new or enlarging T2 lesions in a 2-year follow-up study).	87 MS	[140]	[11]
anti-virus Ab	anti-MRZR Ab	anti-MRZR Ab	Antibodies against Measles, Rubella and Varicella Zoster	CSF	disease severity	Measles virus antibody index was associated with increased EDSS and Gd-enhancing lesions (p=0.031)	68 (61 RR, 7 CIS)	[141]	[13]
					CIS to MS	In a 2 years follow-up longitudinal study, the presence of anti-MRZR IgG was significantly more frequent in patients who converted to clinically defined MS (p=0.04).	89 CIS	[142]	[13]
autoantibodies	anti-NFL Ab	anti-NFL Ab	Antibodies directed against Neurofilament Light chain	CSF	CIS to MS	Ratio of CSF anti-NFL/serum anti-NFL antibodies is significantly higher in CIS converted to clinically definite MS (p<0.005, 3 years follow-up).	39 CIS	[103]	[12]
				serum	progression	NFL Ab correlate with clinical disability and progressive disease course	?	[143]	[11]
autoantibodies	anti-α tubulin Ab<br>anti-β tubulin Ab	anti-α tubulin Ab<br>anti-β tubulin Ab	Antibodies directed against cytoskeletal components	CSF	disease severity	The CSF anti-tubulin index correlates significantly with EDSS in one study [144]<br>No correlation was observed in other studies [144, 146]	39 RR, 10 PP, 18 SP [144];<br>35 MS [145];<br>47 MS [146]	[144-146]	[11]
Other Ab	anti-CSF114(Glc)	anti-CSF114(Glc)	Antibodies against a synthetic glycopeptide	serum	progression	Serum antibody titer parallels clinical activity and brain lesions positive to MRI, suggesting that it may have a high prognostic value in monitoring disease progression (6 months follow up on RRMS with EDSS <3.5)	40 RR	[147]	[11]
Ig light chains	k FLC	Immunoglobulin free light chain K	Immunoglobulin free light chain K concentration	CSF	CIS to SM	-The group with higher concentration of kFLC had a higher probability of conversion to clinically definite MS (p<0.0001) [148];<br>-The group of patients who converted to clinically definite MS had a higher median of CSF k concentration compared to those that remained stable (0.45 mg/L vs. 0.11 mg/L, p<0.0001) [149]	78 CIS [148];<br>141 CIS [149]	[148, 149]	[24, 28]
				CSF, serum	CIS to SM	-Patients with F20a higher FLC quotient ([CSF kFLC]/[serum kFLC]) was higher in CIS patients who converted to MS (n=39) than in those who didn't (n=38)<br>-The kappa quotient was similar but not superior to OCB in predicting conversion from CIS to MS (kappa quotient: sensitivity=86.8%, specificity=38.5%, positive predictive value (PPV)=57.9%. OCB: sensitivity=89.1%, specificity=33.3%, PPV=57.4%)	77 CIS	[150]	[24, 28]

	k FLC - K index	Immunoglobulin free light chain K	Immunoglobulin free light chain K, measured with K index: (CSF-kFLC * serum-albumin)/(serum-kFLC * CSF-albumin)	CSF, serum	CIS to SM	Patients with high kappa index (>10.62) showed a greater probability of conversion to MS (Kaplan-Meyer survival analysis, p<0.0001, follow-up: 2 years).	334 CIS	[36]	[36]
	K FLC/ L FLC ratio	Immunoglobulin free light chain K		CSF	CIS to SM	A lower kappa FLC/ lambda FLC ratio has been associated with a higher risk of conversion (HR=2.89, 95% CI=1.17-7.14; p<0.05) [151]<br>A lower kappa/lambda ratio was observed also in other studies [152]	61 CIS [151]<br>43 CIS [152]	[151, 152]	[30, 35]
	K FLC/ L FLC ratio	Immunoglobulin free light chain K		CSF	disease severity	The sensitivity and specificity of a CSF κ:λ FLC ratio at <9.0 for predicting an EDSS of ≥3.0 at the 5-year follow-up period was 75.0% and 57.1%, respectively. Neither kappa nor lambda by themselves were significantly associated with disability progression.	43 CIS, 50 RR, 20 PP.	[152]	[35]
	k FLC	Immunoglobulin free light chain K	Immunoglobulin free light chain K	CSF	disease severity	-Rudick et al. studied 5 prognostic factors (myelin basic protein, IgG synthesis rate, IgG index, kFLC and λFLC) and concluded that CSF kFLC were the best predictors of physical deterioration (assessed with EDSS, ambulation index, 9-hole peg test and box and blocks test). [153]<br>-Elevated CSF free kappa light chains (≥ 1.53 μg/mL) predicted progression to need for ambulatory assistance within 10 years (PPV = 66.7%) or over the whole course of disease (PPV = 88.9%, mean follow-up: 15 years) and likelihood of reaching a MSSS > 6 (specificity 87.5%, positive predictive value 88.9%). [154]<br>-Conversely, Desplat-Jégo found no correlation between kFLC indices and disease progression (judged by: time to conversion to MS, number of relapses, number of relapses/length of follow-up, EDSS score change). [155]	36 MS [153]<br>57 MS [154]<br>15 CIS [155]	[153-155]	[12, 28]

TABLE S4: Chitinases / chitinase-like proteins

biomarker description	biomarker subtype	full name	body fluid	outcome	observations	n of patients	primary studies	review
Chitin-binding proteins homologous to chitinases, but lacking the capacity of chitin hydrolysis	CHI3L1	Chitinase 3-like 1	CSF	CIS to MS	Prognostic CSF biomarker of conversion from CIS to MS:<br>-Increased concentration of CHI3L1 correlates with higher likelihood of conversion from CIS or optic neuritis to clinically definite MS.<br>-Elevated CHI3L1 levels correlate with shorter time to convert from CIS to MS and more rapid development of disability	813 CIS [75];<br>50 CIS [157];<br>78 ON [158]	[75, 156-158]	[17, 26, 31]
			CSF, serum	CIS to MS	CIS patients with higher CHI3L1 CSF and serum levels had higher conversion rate and shorter conversion time to definite MS (p<0.01 for CSF, p<0.001 for serum).	?	[156]	[23, 25, 38]
			CSF	CIS to MS	Longitudinal study.<br>-CHI3L1 levels were higher in CIS patients who converted to clinically definite MS (p=8.1x10-11), independently of brain MRI abnormalities and presence of IgG oligoclonal bands (p= 3.7x10-6).<br>-Elevated CHI3L1 levels correlate with shorter time to convert from CIS to MS (p=5.6x10-9).	813 CIS	[75]	[3, 13, 21, 23, 25]
					-Only chitinase 3-like 1 was validated and cerebrospinal fluid levels were increased in patients who converted to clinically definite multiple sclerosis compared with patients who continued as clinically isolated syndrome (P = 0.00002).<br>-Time to clinically definite MS was shorter in patients with high CHI3L1 levels (p=0.003).	60 CIS (screening)+<br>84 CIS (validation)	[76]	[13, 18, 21, 23]
			CSF	disease progression in CIS	CHI3L1 CSF level is a strong predictor of disability progression:<br>In a prospective study, levels above the 170 ng/ml cut-off conferred a 4-fold increased risk for the development of disability and were associated with earlier disability progression (5 years difference in mean time at EDSS 3.0) (p1.8x10-10).	813 CIS	[75]	[13, 21, 25]
			CSF	progression	CHI3L1 levels significantly correlated with the number of gadolinium enhancing lesions (Spearman r=0.32, 0=0.037) and the number of t2 lesions (Spearman r=0.44, 0=0.003) in brain at baseline.	44 definite MS	[76]	[38]
					CHI3L1 levels were higher in patients with active disease and correlated with the number of gadolinium enhancing lesions and EDSS score (Spearman r = 0.32, p = 0.0089).	82 RRMS<br>35 SPMS	[159]	[49]
					High CSF levels were associated with earlier disability progression (reaching EDSS = 3 (HR (95% CI): 2.78 (1.48 - 5.23); p = 0.001) and EDSS=6 (HR (95% CI): 4.57 (1.01 - 20.83); p=0.05).	109 CIS<br>192 RR	[63]	[13, 23]

TABLE S5: cytokines, chemokines, TNF-receptor superfamily members, biomarkers of innate immunity

biomarker class	biomarker subtype	full name	biomarker description	body fluid/tissue	outcome	observations	n of patients	primary studies	review
Chemokine	CCL11	Eotaxin-1	Eosinophil attractant and ligand for T cells CCR5 receptor	CSF, plasma	disease duration	Protein levels correlates with duration of disease with an estimated increase per year of 1.1% (CSF) and 1.9% (plasma) (PCSF = 3.5×10^{-5}, Plasma = 3.11×10^{-5}).	136 MS	[160]	[49]
TNF-receptor superfamily	Fas<br>FasL	Fas cell surface death receptor	Members of the TNF-receptor superfamily, central role in the physiological regulation of programmed cell death	PBMC	progression	Fas and FasL predict slower long-term disability progression:<br>-High levels of Fas mRNA were associated with favorable disease course in RRMS (low EDSS) ($R2 = 0.74$, $P = 0.0001$, $n = 13$)<br>-High levels of FasL were associated with milder disease progression in SPMS ($R2 = 0.86$, $P = 0.0001$, $n = 12$)<br>Follow-up: 10 years	25 (13 RR, 12 SP)	[161]	[11]
TNF-receptor superfamily	FasL	Fas cell surface death receptor ligand		PBMC	disease activity	Increased levels of FasL in RRMS, especially in remission ($p=0.0002$) [162]<br>In RRMS patients FasL mRNA level was increased prior to the exacerbations and decreased during clinical activity (14 relapses, $p=0.012$) [163].	47 MS [162];<br>13 (8RR, 5 SP) follow-up: 18 month [163];	[162, 163]	[11, 25]
TNF-receptor superfamily	Fas	Fas cell surface death receptor	Member of the TNF-receptor superfamily, central role in the physiological regulation of programmed cell death	PBMC	disease activity	Increased mRNA expression of Fas has been reported to be associated to disease activity:<br>-Fas mRNA level increased during clinical activity (14 relapses, $p<0.05$) [163].<br>-Serum and CSF sFas (serum Fas) levels higher in patients with active disease compared to patients in remission ($p<0.005$) [164].<br><br>-The serum sFas level was statistically higher in active RRMS patients than in stable RRMS ($P=0.03$) [165]<br>In active RRMS patients the median of CD4+ Fas+ to total CD4+ and CD8+ Fas+ to total CD8+ from relapse-related measurements were higher than the median from relapse-unrelated measurements ($P=0.003$, 0.004, respectively) [165]<br>Increased levels of sFas in patients with worsening EDSS and accumulation of lesions [166]	13 (8RR, 5 SP) follow-up: 18 month [163];<br>51 MS [164];<br>16 RR follow-up: 1 year [165];<br>72 MS, 17 CIS follow-up: 1 year [166]	[163-166]	[11, 25]
Cytokines	TNFa<br>IL1b<br>RANKL		proinflammatory cytokines		disease activity	Correlated with onset of relapses: the two cited primary studies report differences in cytokine secretion in patients with active compared to stable MS [167] or in patients with younger age of onset [168]	18 MS [167];<br>72 MS [168]	[167, 168]	[11]
Cytokines	TNFa<br>CCL2		proinflammatory cytokines	serum	disease activity	Increased levels seem to reflect inflammatory responses especially in PPMS (needs evaluating in long term studies involving large cohorts)	72 MS<br>17 CIS	[166]	[25]
Cytokines	TIM-3	T-cell immunoglobulin and mucin	Th1-specific cell surface protein that regulates macrophage activation, inhibits Th1-mediated	PBMC	disease progression	-TIM-3 levels were lower in patients that converted to SPMS ($n=20$) compared to those that remained RR ($n=37$) after 10 years of follow-up ($p<0.02$).	57 MS	[169]	[45]

		domain containing 3	auto- and alloimmune responses, and promotes immunological tolerance.			-Patients with lower TIM-3 expression reached EDSS = 6 earlier (p<0.005)			
Cytokines	TNFa	tumor necrosis factor alpha	proinflammatory cytokines	T cells	severity	A correlation was found between the percentage of tumor necrosis factor-alpha-producing CD4(+) T cells at baseline and the change in T2 lesion load during 3-year follow-up (r = 0.79, adjusted r(2) = 0.59, p = 0.001)	14 SPMS	[170]	[9]
Chemokines	CXCL13	chemokine (C-X-C motif) ligand 13	The chemokines and their receptors play an important role in the recruitment of autoreactive immune cells to the CNS and are present in MS plaques	CSF, serum	disease activity	CXCL13 is up-regulated in patients with active disease:<br>- There was a significant positive correlation between the pattern of serum levels of CXCL13 and MRI activity during the first (r = 0.33, p < 0.05) and the full 2 years (r = 0.35, p < 0.01) of the study [171].<br>-CXCL13 CSF concentration correlated with leukocyte count (rho=0.69, p<0.001) and B-cell count (rho=0.83, p<0.001), with markers of immune activation and disease activity in CIS and RRMS [172].<br>-There was a significant and positive correlation between CXCL13 CSF level and relapse rate, EDSS and the number of lesions detected by MRI [79].<br>-CXCL13 level was higher during relapse (P=0.02) [173].	74 RR [171];<br>22 CIS, 50 RR [172]<br>466 MS: 79 CIS, 323 RR, 40 SP, 24 PP [79];<br>64 MS [173]	[79, 171-173]	[13, 23, 25, 45]
				CSF	CIS to SM	CIS patients that converted to MS within 2 years of initial diagnosis had significantly higher levels of CSF CXCL13 than those that remained in CIS:<br>-p<0.001 for studies [79] and [174].<br>- 49.9 pg/ml versus 16.3 pg/ml; p < 0.0001 [175]	79 CIS [79];<br>91 CIS [174];<br>110 CIS [175];	[79, 174-175]	[12, 13, 23, 49]
Cytokines	IL6	Interleukin 6	proinflammatory cytokines	serum	disease activity	Positively correlated with relapse frequency in female patients (r2=0.511, P=0.009)	39 MS	[176]	[11, 49]
Cytokines	PTX3	Pentraxin 3	component of innate immune system	plasma	disease activity	Significantly increased during relapse stage (p<0.001)	22 MS	[177]	[11]
Cytokines	OPN	Osteopontin	Play a role in the migration of macrophages and DC to sites of inflammation. It is highly expressed within MS lesions and is significantly higher in the blood and CSF of MS patients compared to controls	CSF, serum, plasma	disease activity	OPN levels are increased during relapses in RRMS, correlated with disease severity and relapse rate and appear just prior to the appearance of Gd-enhancing lesions:<br>-cross-sectional study, OPN plasma level was elevated in RR patients who went into relapses in 3 months before/after OPN evaluation compared to RR in relapse phase (p=0.019) [178]<br>- phase II clinical trial (17 months follow-up): OPN serum levels were increased 1 months prior to increase in MRI Gd-enhancing lesions count (p=0.027) [179]<br>- cross sectional, CSF levels increased during attacks [180]<br>-cross sectional study, OPN plasma levels were higher in RR patients who went into relapse (p=0.002) [181]<br>-5 years follow-up study: CSF OPN levels were higher in samples collected during relapse than during remission (p=0.010) [181]<br>-The association with disease severity was not reported by two studies on plasma [183, 184].	30 RR [178];<br>16 RR [179];<br>25 CIS, 41 RR, 9 PP, 28 SP [180]<br>221: 115 RR, 71 PP, 35 SP [181]<br>74 MS (32 RR) [182];<br>492 MS [183];	[178-184]	[11, 13, 16, 23]

Immune cells surface markers	CD80	CD80	Th1 stimulatory molecule, member of the B7 family of costimulatory molecules	T cells	disease activity	Levels of CD80 increase during active disease	11 MS	[185]	[11]
Immune cells surface markers	CD86	CD86	Immunoregulatory member of the B7 family of costimulatory molecules	T cells	disease activity	Levels of CD86 decrease during active disease	11 MS	[185]	[11]
Costimulatory molecules	PD-1 PD-L1	Programmed death 1; Programmed death 1 ligand	PD-1/PD-L1 interaction inhibits T-cell proliferation and cytokine production, maintaining immune tolerance	Lymphocytes, macrophages	disease activity	Proposed as biomarker because increased expression of PD-1 and PD-L1 is associated with disease remission: PD-1: (CD4+, p = 0.04; CD8+, p = 0.002); PD-L1: p = 0.066	78 RR (40 in relapse, 28 in remission)	[186]	[11]
Cytokines	Survivin	Survivin	Inhibitor of apoptosis	T cells	disease activity	Increased during active disease	?	[187]	[11]
TNF-receptor superfamily	IRF8	Interferon 8	IRF8 is a transcription factor of the interferon (IFN) regulatory factor (IRF) family	plasma, CSF	disease subtype	Screening: 3450 genes; target 1: 334 genes; target 2: 43 genes. Significant differenced were observed for IRF8 between CIS and RRMS or between CIS and SPMS with all four datasets	26 (screening); 172 (target 1); 443 plasma + 573 CSF (target 2); 50 plasma (IRF 8 verification)	[188]	[18]
Cytokines	LAG-3	Lymphocyte activation gene 3	Cell-surface protein belonging to the Immunoglobulin superfamily, expressed in several cell types. It negatively regulates proliferation and activation of T-cells	PBMC	disease progression	-Patients with lower LAG-3 expression reached EDSS = 6 earlier (p<0.005)	57 MS	[169]	[45]
Cytokines	BAFF	B-cell-activating factor	Cytokine and drug target B-cell-activating-factor	plasma	disease activity	Longitudinal study: BAFF levels were higher in patients in stable patients without relapses compared with relapsing patients.	170 RR	[189]	[17]

TABLE S6: miRNA

biomarker subtype	full name	proposed pathway/target	body fluid	outcome	observations	n of patients	primary studies	review
let-7 miRNA	let-7 miRNA family	potent activators of toll-like receptor signaling in macrophages and microglia, regulating stem cell differentiation and neurogenesis	plasma	RR to SP	Circulating let-7 miRNA differentiate RRMS from SPMS. Let-7c and let-7d are downregulated in SPMS vs. RRMS. (368 miRNA analyzed)	10 RR, 9 SP	[190]	[11, 52]
miR-18b	miR-18b	PERQI, GABI, SIM2, GLRB, REXO2, BTG3, HSF2, MDGAI, UBTD2, TSHZ3, C7orf42, HMBOXI, CLIP3, UBE2Z	PBMC	disease activity	increased during relapses compared to patients in remission (364 miRNA analyzed)	47 MS	[191]	[14]
miR-27a	miR-27a	Regulates the differentiation of Th1 and th17 cells	Serum	RR to SP	Downregulated in SPMS vs. RRMS	29 RR, 19 SP	[192]	[52]
miR-92a-1	miR-92a-1	CD40-CD40L pathway Regulates cell cycle and Th1 differentiation	serum	disease severity	Correlates with increasing disease severity disability, down regulated in SPMS vs. RRMS (368 miRNA analyzed)	10 RR, 9 SP [190] 37 MS [193]	[190, 193]	[25, 42, 52]
miR-107 miR-146b	miR-107 miR-146b-5p		Peripheral blood leukocytes	disease severity	Positive correlation with EDSS. Pearson correlation coefficients: miR-107 = 0.57, miR-146b-5p = 0.60	34 untreated RRMS	[194]	[42]
miR-874 miR-3614	miR-874 miR-3614		Peripheral blood leukocytes	disease severity	Negative correlation with EDSS. Pearson correlation coefficients: miR-874 = -0.53, miR-3614 = -55	34 untreated RRMS	[194]	[42]
miR-150	miR-150		CSF	CIS to MS	Higher miR-150 levels in CSF of CIS patients that converted to MS after a median follow-up of 52 months compared to those who did not convert ($p < 0.0001$) The ratio of miR-150/miR-204 has an AUC=0.775 for differentiating CIS patients who converted from patients who did not convert.	96 CIS	[195]	[37, 42, 43, 49]
miR-155	miR-155	promotes inflammatory properties of T cells and Th17 and Th1 cell differentiation	PBMC	disease severity	Expression correlates with disease severity (both MS patients and EAE mice). Upregulated in active compared to inactive regions	31 MS	[196]	[11, 37]
miR-181c	miR-181c	MeCP2, XIAP, HMGA1, GDNF, VEGF. Regulates neuronal maturation and cortex synaptogenesis	CSF	CIS to MS	High levels of CSF miRNA-181c were independently associated with conversion from CIS to RRMS in multivariate Cox regression analysis (hazard ratio 2.99, 95%, P=0.005)	58 CIS	[197]	[30, 37, 43]
miR-181c miR-633	miR-181c miR-633		CSF	RR to SP	miR-181c and miR-633 could be used to differentiate relapsing-remitting (RRMS) and secondary progressive MS (SPMS) courses with a specificity of 82% and sensitivity of 69% (These miRNA levels were increased in RRMS when compared to SPMS).	53 MS	[198]	[14, 20, 45, 52]
miR-193a	miR-193a		PBMC	disease activity	increased in remission (364 miRNA analyzed)	47 MS	[191]	[14]
miR-326	miR-326	silencing of miR-326 in mice cause fewer Th17 cells and milder EAE	PBMC	disease severity	Expression correlates with disease severity (both MS patients and EAE mice) [199]	21 brain lesions [200]	[199, 200]	[11, 14]

					Increased expression in active lesions but not in inactive lesions. Analysed 365 miRNAs [200]			
miR-337	miR-337	Rap1 signaling	serum	disease severity	-Negative correlation with EDSS in three out of four independent cohorts: Spearman correlation = -0.41, p-value = 0.007<br>-Downregulated in SPMS vs. RRMS (p=0.010)	115 RR, 51 SP	[201]	[37, 42, 52]
miR-454	miR-454		serum	disease severity	Correlates with increasing disease severity disability<br>(368 miRNA analyzed)	10 RR, 9 SP [190]<br>37 MS [193]	[190, 193]	[25, 42]
miR-572	miR-572	NCAM1, SEPT8, TAOK2, QRICH2	serum	disease activity	-mir-572 expression was significantly upregulated in SPMS and in RRMS during disease relapse<br>-miR-572 expression correlated with EDSS scores (R Spearman = 0.491; p < 0.05) independently of the clinical phenotype	31 RR, 15 SP, 16 PP	[202]	[26, 45]
miR-599	miR-599	L4RC4C, ZSWIM6, NFIA, ROCKI, TGFB2, ATMIN	PBMC	disease activity	increased during relapses compared to patients in remission.<br>(364 miRNA analyzed)	47 MS	[191]	[14, 26]
miR-922	miR-922	UCHLI; APHIA, UCHLI, CLIC5, STX17, RNF2, HIFIAN	CSF, serum	CIS to MS	miR-922 expression was higher in CIS patients who converted to clinically defined MS compared to non-converters (CSF: p=0.027, serum: p=0.048). 1 year follow-up	58 CIS	[197]	[26, 49]

TABLE S7: Cholesterol and markers of cholesterol turnover

biomarker class	biomarker subtype	full name	body fluid	outcome type	outcome	observations	n of patients	primary studies	review
lipoproteins	LDL	Low density lipoprotein	serum	severity	EDSS	A positive correlation was found between LDL levels and EDSS:<br>-(p=0.007, ANOVA) [203]<br>-(p=0.050, Pearson correlation) [204]<br>-p=0.006, linear regression [205] (follow-up: 2.2 years)	84 RR, 16 SP [203];<br>30 RR [204];<br>395 RR, 82 SP, 15 PP [205]	[203-205]	[22]
lipoproteins	LDL	Low density lipoprotein	plasma (a), serum (b)	severity	lesions	-A direct correlation was found between LDL levels and the number of contrast-enhancing lesions (CEL) (p=0.02), 6 months follow-up [206]<br>-Higher LDL levels are associated with the development of new T2 lesions (p=0.006) and with the number of new or enlarging of T2 lesions (p=0.008), 2 years follow-up [207]	18 CIS [206];<br>135 CIS [207]	[206-207]	[22]
lipoproteins	LDL	Low density lipoprotein	serum	severity	visual outcomes	-LDL levels > 100 mg/dl are associated with reduced RNFL thickness (p=0.022) and with longer PRVEP (pattern reversal visual-evoked potential) latency (p=0.017). No association with visual acuity	121 RR, 11 SP, 4 PP	[208]	[22]
oxysterols	ox-LDL	oxidized low density lipoprotein	serum, plasma	severity	EDSS	A positive correlation was found between ox-LDL levels and EDSS:<br>-(p=0.011, Pearson correlation)	30 RR	[204]	[22]
oxysterols	24-OHC	24-hydroxycholesterol	plasma	severity	EDSS	An inverse correlation was observed between 24-OHC levels and EDSS (p<0.05)	77 RR, 15 SP, 3 PP	[209]	[22]
oxysterols	24-OHC	24-hydroxycholesterol	CSF	severity	lesions	Both 24-OHC and 27-OHC levels were higher in patients positive for contrast-enhancing lesions (CEL) (p<0.001, 32 CEL positive, 56 CEL negative)	88 RR	[210]	[22]
oxysterols	24-OHC	24-hydroxycholesterol	serum	severity	brain volume	Normalized brain volume inversely correlates with serum 14-OHC levels (p=0.004)	51 RR	[211]	[22]
cholesterol precursors	Lanosterol	Lanosterol	CSF	severity	lesions	Positive correlation between lanosterol levels and T2 lesions load (p<0.03)	51 RR, 39 SP, 15 PP	[211]	[22]
lipoproteins	HDL	High density lipoprotein	serum	severity	lesions	Higher HDL levels were associated with reduced CEL presence (p=0.001) and CEL (contrast-enhancing lesions) volume (p<0.001)	395 RR, 82 SP, 15 PP	[205]	[22]
lipoproteins	HDL	High density lipoprotein	serum	severity	visual outcomes	-Higher levels of HDL correlate with thinning of retinal nerve fiber layer (RNFL) on optical coherence tomography (OCT): p=0.008 and with longer PRVEP (pattern reversal visual-evoked potential) latency (p=0.043)<br>-HDL levels > 60 mg/dl are associated with reduced RNFL thickness (p=0.001)	121 RR, 11 SP, 4 PP	[208]	[22]
lipoproteins	ApoB	Apolipoprotein B	serum	severity	EDSS	Higher ApoB levels are associated with increased EDSS at baseline (p=0.003)	149 RR, 20 SP, 9 PP	[212]	[22]
lipoproteins	ApoB	Apolipoprotein B	serum	severity	lesions	High ApoB levels were associated with increased number of new T2 lesions (p<0.001) and increased number of new or enlarging T2 lesions (p<0.001), 2years follow-up.	181 CIS	[213]	[22]
lipoproteins	ApoE	Apolipoprotein E	serum	severity	brain volume	-Higher ApoE levels were associated with greater grey matter atrophy (p<0.001), 2 years follow-up [213]<br>-ApoE increase and PLP (phospholipid transfer protein) decrease was associated with increased normalized brain volume. No significant trend with ApoE itself	181 CIS [213];<br>39 RR, 37 SP, 15 PP [214]	[213, 214]	[22]
lipoproteins	ApoE	Apolipoprotein E	serum, CSF	disease activity	relapses	ApoE levels were higher in patients in relapse (p<0.001, cross-sectional study)	33 RR	[214]	[22]

cholesterol	total cholesterol	total cholesterol	serum	severity	EDSS	A positive correlation was found between total cholesterol levels and EDSS: -p=0.01, ANOVA [203] -p=0.027, Pearson correlation [204] -p=0.001, follow-up: 2.2 years [205] -p=0.037, follow-up: 2.2 years [212]	84 RR, 16 SP [203]; 30 RR [204]; 395 RR, 82 SP, 15 PP [205]; 149 RR, 20 SP, 9 PP [212]	[203-205, 212]	[22]
			serum	severity	MSSS	A positive correlation was found between total cholesterol levels and MSSS: p=0.008	395 RR, 82 SP, 15 PP	[205]	[22]
			plasma (a), serum (b)	severity	lesions	-A positive correlation was found between total cholesterol levels and the number of contrast-enhancing lesions (p=0.011) [206] -Higher total cholesterol levels are associated with the development of new T2 lesions (p=0.001, 2 years follow-up) [207] -Total cholesterol levels > 200 mg/dl are associated with the development of new T2 lesions (p=0.001) and with the number of new or enlarging of T2 lesions (p=0.001), 2 years follow-up [207]	18 CIS [206]; 135 CIS [207]	[206, 207]	[22]
			serum	severity	RNFL thickness	-total cholesterol levels > 200 mg/dl are associated with reduced RNFL thickness (p=0.001)	121 RR, 11 SP, 4 PP	[208]	[22]
cholesterol	TC:HDL	total cholesterol HDL	serum	severity	EDSS	Elevated total cholesterol / HDL ratio is associated with subsequent worsening of EDSS after a mean of 2.2 years (p=0.029)	149 RR, 20 SP, 9 PP	[212]	[22]
triglycerides	triglycerides	triglycerides	serum	severity	lesions	Increased triglycerides concentration is associated with the presence of contrast-enhancing lesions (p=0.038)	395 RR, 82 SP, 15 PP	[205]	[22]
phospholipids	LPA-18:2	Lyso-phosphatidic acid C18:2	plasma	disease progression	EDSS, MRI lesions	LPA-18:2 was significantly less abundant in PPMS patients with a rapidly deteriorating disease course compared with those with mild progression, and its levels inversely correlated with the severity of the neurological deficit (r = − 0.59; *p* = 0.009).	19 PPMS, 11 SPMS	[216]	[53]

TABLE S8: Oxidative stress biomarkers

biomarker class	biomarker subtype	full name	biomarker description	body fluid	outcome	observations	n of patients	primary studies	review
Metabolites	Lactate	Lactate	Produced during anaerobic energy metabolism. Impaired mitochondrial function in MS may cause an increase of these metabolites	serum	disease activity	Positive correlation between serum lactate levels and EDSS ($R2=0.419$; $p<0.001$)	613 MS	[217]	[11]
Reactive oxygen species	NO	Nitric oxide	Free radicals contribute to the neurodegenerative cascade in the CNS and are increased in acute demyelinating lesions	serum, plasma	disease severity	-A phase II clinical trial found that higher tNOx levels significantly correlate with lower relapse rates over an 18-month period [220].<br>-No relationship was observed with EDSS [218] or disease duration [219] (cross- sectional studies)	170 MS [218]<br>23 MS [219]<br>23 MS: 13 RR, 16 SP [220]	[218-220]	I[19]
				CSF	disease activity	-Association between tNOx and the number of gadolinium-enhancing lesions (24 MS during relapse and remission) [221]<br>-Association with the volume of gadolinium-enhancing lesions ($p=0.01$) [222].<br>-Cross sectional studies reported higher tNox in patients with acute relapsing disease compared with those with stable disease [223, 224]<br>-One study reports higher tNox immediately following relapse when compared to remission phase [225]<br>-A study reports a negative correlation between post relapse NO levels and the EDSS decrease at 8 weeks, however the review authors suggest that this result should be interpreted with caution due to the short time range. [226]<br>-Studies that fail to identify a relationship between NO and disease activity: Milijkovic 2002 [227]	24 MS [221]<br>51 MS: 20 RR, 21 SP, 10 PP [222]<br>17 MS [224]<br>8 remission, 7 exacerbation[225]<br>34 RRMS in exacerbation [226]<br>50 MS [227]	[221-227]	[19, 25]
				CSF	progression	-Higher baseline CSF tNOx in patients with EDSS disability progression when compared to stable disability ($p=0.02$) (longitudinal study, 3±0.5 years follow up) [222]<br>-Higher mean level of CSF nitrate in RRMS patients than in patients with progressive disease [228]<br>-These studies failed to observe a significant relationship with disease subtype [229] and disability [230].	34 MS [222]<br>61MS [228]<br>35 MS: 15 RR, 10 SP [229]<br>105 MS [230]	[222, 228-230]	[19]
				Urine	disease subtype	Elevated tNOx/creatinine quotient in patients with CIS or RRMS when compared to progressive forms ($p=0.006$) (case/control study)	129 MS: 23 CIS, 46 RR, 60 progressives	[231]	[19]
Reactive oxygen species enzymes	SOD	Superoxide dismutase	Enzymatic detoxification of superoxide is through the actions of SODs and peroxidases. SODs are implicated in MS	Blood	disease subtype	-A cross-sectional study observed higher SOD activity in CIS compared to RRMS ($p<0.05$) [232]<br>-However another study measured platelet SOD1 and SOD2 activity in MS and found no significant difference between groups [233]	50 CIS, 57 RR [232]<br>30 MS [233]	[232, 233]	[19]

			pathogenesis as part of the physiological response to oxidative stress.	CSF	disease severity	Negative correlation between SOD activity and EDSS (p<0.01)	50 CIS, 57 RR	[234]	[19]
Reactive oxygen species enzymes	CAT	Catalase	Major intracellular peroxidase, highly expressed in active demyelinating plaques and grey matter astroglia in MS	CSF, plasma	disease activity	Patients with lower EDSS had higher CSF and plasma catalase activity	50 CIS, 57 RR	[234]	[19]
End-products of oxidation	3-NT	3-nitrotyrosine	Peroxynitrous acid, a highly reactive ROS, reacts with tyrosine residues to form nitrotyrosines. Such modification may alter protein conformation and cause pathogenic effects	plasma	disease subtype	3-NT plasma level was 2-fold higher in SPMS tan in RRMS patients (p<0.01)	10 RR, 10 SP	[235]	[19]
Products of lipid peroxidation	8-epi-PGF2alpha	Isoprostanes	Prostaglandin F-like products of cyclooxygenase-independent peroxidation of fatty acids.	CSF; (plasma)	disease severity	-A study reported that CSF levels of 8-epi-prostaglandin (8-epi-PGF2alpha) were correlated with degree of disability [236].<br>-However in a successive study the same group reports that CSF levels of 8-epi-PGF2alpha are not correlated with gadolinium enhanced lesions and time since relapse [237].<br>-However a longitudinal study observed no evidence to support a prognostic role of plasma levels of 8,12-iso-iPFalpha with regards to: conversion to definite MS, EDSS score, or MRI outcome [238].	41 RR [236, 237];<br>17 CIS, 41 RR, 5 PP [238]	[236-238]	[19]
Products of lipid peroxidation	MDA	Malondialdehyde	Secondary aldehyde formed in the reaction between ROS and polyunsaturated lipids. It can form deleterious adducts with protein and DNA.	CSF, plasma	disease severity	CSF and plasma MDA levels were higher in patients with higher EDSS score (p<0.05)	57 RR, 50 CIS	[234]	[19]
				CSF, serum	disease activity	MDA levels were higher in relapse than in remission	37 RR	[239]	[19]

TABLE S9: Immunoprofile

cell type	cell subpopulation	marker	body fluid	outcome	observations	review
T cells	CD3+		CSF	disease activity	increased in relapses vs. remission	[5]
Th1 cells	CCR5+, CXCR3+		peripheral blood	disease activity	Th1/Th2 ratio correlates with disease activity	[5]
Th17 cells	CCR6+		peripheral blood	disease activity	increased in relapses vs. remission	[5]
CD8+ Treg cells	CD25+ FoxP3+ CD28-		CSF	disease activity	decreased during relapses vs. remission	[5]
T memory cells		CD25	CSF	disease activity	increased in relapses vs. remission	[5]
T memory cells		CD25	peripheral blood	disease activity	increased in relapses vs. remission, correlation with MRI activity	[5]
T memory cells		MHC-II	peripheral blood	disease activity	decreased prior to new MRI lesion formation	[5]
T memory cells		HLA-DR	CSF	disease activity	decreased during relapses vs. remission	[5]
B cells	CD19+ CD20+		CSF	disease progression	correlation with disease progression	[5]
B memory cells		CD5	peripheral blood	disease activity	correlation with MRI activity	[5]
B memory cells		CD5	peripheral blood	CIS to MS	predicts CIS to MS conversion	[5]
B memory cells		CD80	peripheral blood	disease activity	increased in relapses vs. remission	[5]
plasma cells	CD20- CD138+		CSF	disease activity	correlation with disease activity	[5]
NK cells		CD95/Fas	peripheral blood	disease activity	decreased during relapses vs. remission	[5]
Monocytes	CD14+		CSF	disease activity	decreased during relapses vs. remission	[5]
plasmacytoid dendritic cells	CD123+, CD303+		CSF	disease activity	increased in relapses vs. remission	[5]
T cells		CCR5	CSF	disease activity	increased in relapses vs. remission	[5]
T cells		CXCR3	CSF	disease activity	correlation with MRI activity	[5]
T cells		CXCR3	peripheral blood	disease activity	correlation with clinical and MRI activity	[5]
T cells		ICAM-1	CSF	disease activity	decreased during relapses vs. remission	[5]
T cells		ICAM-1	peripheral blood	disease activity	negative correlation with MRI activity	[5]
monocytes		ICAM-1	CSF	disease activity	decreased during relapses vs. remission	[5]
T cells		ICAM-3	CSF	disease activity	decreased during relapses vs. remission	[5]
monocytes		ICAM-3	peripheral blood	disease activity	negative correlation with MRI activity	[5]

TABLE S10: Extracellular vesicles

EV marker	Cell origin	body fluid/tissue	outcome	observations	n of patients	primary studies	review
CD31+ endothelial microparticles	Endothelial cells	plasma	disease activity	CD31+EMV were higher in relapse and returned to nearly control value during remission, thus they could be a marker of exacerbation.	50 MS	[240]	[11, 47, 50]
CD4+/CCR3+ (Th2 cells) and CD4+/CCR5+ (Th1 cells)	Th1and Th2 lymphocytes	CSF	disease activity	The presence of lesions in the brain and spine on gadolinium-enhanced MRI was associated with an increase in the numbers of vesicles	39 MS	[241]	[47, 50, 51]
CD19+/CD200+	Naïve B cells	CSF	disease activity	Significant (p = 0.030) reduction in the number of vesicles in patients in relapse phase	39 MS	[241]	[47, 51]
CD11b/c	microglia	CSF	disease activity	Levels peak at disease onset and during relapses and decrease in the chronic phase of the disease.	28 RR, 28 CIS	[242]	[47, 51]
Exosomes expressing myelin proteins		serum	disease activity	Exosomal content of MOG strongly correlated with disease activity and was highest in RRMS patients in relapse and in SPMS patients.	45 RR, 30 SP	[243]	[50, 51]
acid sphingomyelinase-(ASM) enriched exosomes		CSF	disease severity	The amount of ASM for exosomes correlates with EDSS at time of lumbar puncture ($P < 0.05$, $r = 0.335$ for Spearman test).	20 MS	[244]	[50, 51]

TABLE S11: other biomarkers

biomarker class	biomarker subtype	full name	biomarker description	body fluid/tissue	outcome	observations	n of patients	primary studies	review
retroviruses	HERV-W/MSRV	Multiple Sclerosis associated retrovirus	Endogenous retrovirus. It is a presumably complete virus, able to form extracellular infectious virions, releases by leptomeningeal cells of MS patients	CSF	disease severity	Positivity for HERV-W/MSRV at MS onset is correlated with a poor prognosis (higher EDSS after 10 years of follow-up, p=0.004)	22 MS	[245-247]	[32]
Transcription factors	TOB1	transducer of ERBB2, 1	Transcription factor critical for repression of T-cell proliferation	naïve CD4+ T cells	CIS to MS	The expression of TOB1 was significantly downmodulated in CIS patients who rapidly converted to MS	37 CIS	[248]	[11]
Metallo-proteases	MMP9<br>TIMP-1	matrix metalloproteinase 9; TIMP metallopeptidase inhibitor 1;	The balance between matrix metalloproteinases and their inhibitors regulates the digestion of extracellular matrix and basement membranes, and, as a consequence, also the migration of white blood cells in the CNS	serum	disease activity	Higher levels of MMP9 or lower levels of TIMP-1 seem to predict the presence of Gd-enhancing lesions (respectively; odds ratio = 3.3, p = 0.008; odds ratio = 2.2, p = 0.086; follow-up: 15 months) and BBB disruption. However, MMP9 and TIMP-1 levels fluctuate between cohorts and are affected by infections, so their use for monitoring the disease is complex.	24 RRMS	[249]	[11]
Metallo-proteases	MMP9<br>TIMP-1<br>TIMP-2	matrix metalloproteinase 9; TIMP metallopeptidase inhibitor 1; TIMP metallopeptidase inhibitor 2	Matrix metalloproteinases are involved in the degradation of extracellular matrix and contribute to the BBB permeability, myelin breakdown and axonal damage.<br>(TIMPs: MMP tissue inhibitors)	CSF, serum	disease activity	MMP9 increase in CSF/serum during relapses and correlates with MRI disease activity:<br>-Patients who developed new Gd+ lesions had higher levels of MMP-9 than patients who did not develop Gd+ lesions (median 351 vs 226 ng/mL, p = 0.049), follow-up 3 years [250]<br>-Serum MMP9 (p<0.001) and CSF (p<0.02) levels were higher in patients with clinical and MRI disease activity [251]<br>-Clinically active RR patients showed a higher MMP-9/TIMP-1 ratio than RR inactive (p=0.006) (TIMPs: MMP tissue inhibitors) [252]	33 SPMS [250]<br>37 RR, 15 SP, 9 PP [251]<br>21 RR [252]	[250-252]	[23, 25]
Metallo-proteases	MMP2<br>TIMP-2	matrix metalloproteinase 2, TIMP metallopeptidase inhibitor 2		serum	disease severity	Serum levels of MMP-2 and MMP-2/TIMP-2 ratio correlated with EDSS (p<0.001) and MSSS (p<0.05)	40 RRMS; 20 SPMS; 27 PPMS	[253]	[45]
Brain specific protein	S100b	S100b	protein synthesized in brain and released in blood when BBB is disrupted	CSF, serum	disease activity	Concentration of S100b in CSF and serum correlated with disease activity. It appears in plasma and CSF also in response to other disease processes, so it is not very specific for BBB damage	28 MS	[254, 255]	[11]

Neuro-trophins	Neuro-trophins	Neuro-trophins	proteins that can stimulate regeneration and promote repair. Expressed in MS lesions	CSF	disease activity	BDNF was significantly increased after relapse (median [range] pg/ml: 2392 [1093–3122] vs 1399 [591.3–2028]; p = 0.0086) [256]<br>Secretion of brain-derived neurotrophic factor (BDNF) by immune cells was positively associated with increased contrast-enhancing lesion volumes (p=0.026) and with higher white matter volume (p=0.027) [257]<br><br>Reported by the review author but not documented: neural cell adhesion molecule ciliary neurotrophic factor has been associated with disease activity	12 MS that relapsed during follow-up (1 year) [256];<br>52 RRMS [257]	[256, 257]	[25]
Neuro-trophins	BDNF	Brain derived neurotrophic factor	Secreted by neurons and immune cells. Suggested role in preventing neural death and neurodegeneration	serum, plasma	disease activity	Concentration of BDNF have been reported to be increased during relapses	29 MS [255];<br>20 RR, 15 SP [258];	[255, 258, 259]	[6, 23]
Neuro-trophins	Orexin-A	Orexin-A	Orexins are highly excitatory neuropeptides. Orexin-A modulates cognitive and motor functions through different pathways including BDNF mediated signaling	serum	disease severity	-Orexin-A levels negatively correlated with disability indexes (the patients with lower levels required more time to perform motor tasks): 9-hole-peg (p=0.027, R=-0.397) and timed 25-foot walk (p=0.035, R=-380).<br>-Orexin-A levels negatively correlated with progression index (p<0.001, R=-0.712).	25 RRMS	[260]	[45]
Tryptophan Metabolism	IDO	Tryptophan depleting enzyme indoleamine 2,3 dioxygenase	Tryptophan Metabolism trough the kynurenine pathway	serum	disease activity	IDO expression increases during relapses (p<0.001)	21 MS in acute phase + 15 in stable phase	[261]	[25]
Channel	K2P5.1 (KCNK5)	potassium two pore domain channel subfamily K member 5	Potassium channel	T cells	disease activity	Expression of K2P5.1 is upregulated during acute relapse.		[262]	[11]
Complement component	Factor H	Factor H	Regulatory factor of the complement alternative pathway. Recognize and binds C3b.	serum	RR to SP	Factor H level increase progressively with disease progression over a 2-year period in patients transitioning from relapsing to progressive form (P = 0.007), while patients with stable RRMS have constant levels (for a period of 1 year).	350 MS	[87]	[29, 45]
					disease activity	Acute relapse was also associated with transiently increased factor H levels (P = 0.009) compared to stable relapsing disease	350 MS	[87]	[9]
Complement component	C3	C3 complement component	Complement factor of the classical pathway	CSF	EDSS	The authors reported that C3 levels correlate with disability (EDSS) and number of brain MRI lesions (p=0.0034, r2=0.17). The highest C3 levels were observed in progressive patients.	48 MS	[263]	[29]
Complement component	SC5b-9	soluble complement C5b9	Complement factor of the classical pathway	CSF	EDSS	SC5b-9 CSF concentration correlated significantly with neurological disability (EDSS) (r=0.55, P=0.003)	31 MS	[264]	[29]

Complement component	RGC-32	Response gene to complement-32	Involved in cell cycle regulation. Its overexpression leads to cell cycle progression from G1/G0 to G2/M	PBMC	disease activity	Levels of RGC-32 and FasL were significantly decreased in patients sampled during relapses when compared to stable patients (RGC-32: $p < 0.0001$, FasL: $p = 0.0206$)	20 stable RR<br>14 relapsing RR	[265]	[29]
Extracellular peptide	Bri2-23	Bri2-23	Extracellular C-terminal peptide cleaved from the neuronal transmembrane protein Bri2	CSF	disease severity	CSF levels of Bri2-23 show association with clinical measure of cerebellar and cognitive function (case/control study dividing patients according to degree of cerebellar dysfunction)	40 MS	[266]	[12]
Glycoprotein of hepatic origin	Fetuin-A	alpha-2-HS-glycoprotein	Serum glycoprotein synthesized by hepatocytes. It is involved in endocytosis, brain development, formation of bone tissue, calcium metabolism opsonization and immune regulatory functions.	CSF	CIS to MS	Levels of fetuin-A are lower in CIS patients who converted to MS in the follow-up period of 2 years ($p=0.03$): 2 sample sets: 16 CIS (8 converters, MALDI-TOF) + 36 CIS (19 converters, ELISA)	16 CIS (a) + 36 CIS (b)	[267]	[12]
					disease activity	Elevated CSF fetuin-A correlated with disease activity in MS. Fetuin-A was markedly elevated in demyelinated lesions and in gray matter within MS brain tissue.	?	[268]	[12]
14-3-3 protein family	14-3-3 protein	tyrosine 3-monooxygenase/tryptophan 5-monooxygenase activation protein theta	Involved in trafficking and cell signaling	CSF	CIS to MS, disease severity	A positive 14-3-3 assay was associated with a shorter time to conversion to clinical definite MS (risk ratio= 4.1; 95% CI: 1.1 to 15) and to reach an EDSS≥2 at the end of follow-up (OR=14.8; 95% CI: 2.86 to 76.8). (a) The 14-3-3 positive group had a shorter time to conversion to CDMS (7 vs 44.8 months), a higher relapse rate (3.1 vs 1.8) and a higher frequency of patients with EDSS ≥ 2.0 at the end of the study (57 % vs 20.5 %) (b, follow-up of a including 47 new patients). The assay had a high specificity (95.8 %) but a relatively low positive predictive value (57.3 %)	38 MS [269];<br>85 (38 from a) [88]	[269, 88]	[12]
Adhesion molecules	sP-Selectin	soluble platelet endothelial cell adhesion molecule 1	soluble forms of adhesion molecules released by endothelial cells, immune cells and platelets	plasma	disease activity	Upregulated during MS exacerbation:<br>In a group of 11 MS patients analyzed in remission and subsequent relapse sP-selectin concentrations were higher during relapses, $p = 0.0098$.	11 MS	[270]	[25]
Adhesion molecules	sPECAM-1	platelet and endothelial cell adhesion molecule 1	soluble forms of adhesion molecules released by endothelial cells, immune cells and platelets	plasma	disease activity	PECAM-1 values are higher during relapses than in remission phase: $p = 0.001$ (11 patients analyzed both in remission and in relapse)	11 MS	[270]	[25]
Adhesion molecules	sICAM1<br>sVCAM	intercellular adhesion molecule 1	ICAM= cell surface glycoproteins which is typically expressed on endothelial cells and cells of the immune system	CSF, serum	disease activity	During relapse periods patients had significantly higher sICAM1 index values (1.76±0.60) than those found during remission periods (1.01±0.44) ($p<0.05$) Serum and CSF sICAM1 levels were increased during relapses, but not significantly.	24 RRMS	[271]	[12]
Adhesion molecules	sVCAM	vascular cell adhesion molecule-1		blood	disease activity	VCAM levels were increased prior to relapses (18 months follow-up)	7 RRMS	[272]	[9]

Adhesion molecules	NCAM	neuronal adhesion molecule	involved in axonal outgrowth, guidance and fasciculation	CSF	disease severity	CSF NCAM levels were negatively correlated with EDSS (r=-0.53, p=0.0001)	67 MS (18 CIS, 29 RR, 14 SP, 6 PP)	[89]	[27]
Albumin	QAlb	Albumin quotient	Albumin cerebrospinal fluid/plasma concentration quotient. It is a biomarker of blood brain barrier disruption	CSF, serum	disease severity	Increased QAlb at clinical onset was associated with enlargement of lateral ventricles (p = .001) and greater whole brain (p = .003), white matter (p < .001), corpus callosum (p < .001), and thalamus (p = .003) volume loss over 48 months. Higher QAlb was associated with higher Expanded Disability Status Scale score over 48 months (p = .002).	182 CIS	[273]	[17]
amino acid	NAA	N-acetyl-aspartate	Amino acid expressed almost exclusively in neurons	CSF	disease severity	Decrease in CSF NAA levels correlated with higher disability and lesion load: EDSS (r = 0.37, p = 0.016), normalized brain volume (r = 0.49, p = 0.001), T2 lesion load (r = 0.35, p = 0.021) and black hole lesion load (r = 0.47, p = 0.002)	46 MS (26 RR, 12 SP, 8 PP)	[274]	[7, 27]
amino acid	glu	Glutamate	major excitatory neurotransmitter in the CNS	CSF	disease progression	Patients with SP MS who had an increase of at least 1 point in the EDSS score in the last 6 months showed glutamate levels that were significantly greater than those measured in the CSF of patients with SP MS without significant changes in the EDSS score in the same period (P<.001).	25 SPMS	[90]	[27]
membrane signal protein	mRAGE	membrane bound receptor for advanced glycation end products	Signal transduction transmembrane protein	PBMC	disease severity	Negative correlation between the percentage of mRAGE positive PBMCs and MSSS (r=-0.39, p=0.04). Negative correlation between the percentage of mRAGE positive monocytes and MSSS (r=-0.58, p=0.001) or EDSS (r=-0.48, p=0.01).	20 RRMS; 8 SPMS	[275]	[45]
myelin proteins	MBP	Myelin basic protein	It is releases in the CSF after myelin damage and is a marker of demyelination	CSF	disease activity	Positive correlation between CSF MBP levels and gadolinium-enhancing lesions.	44 RRMS, 20 SPMS	[276]	[27]
kyneurines	QA/KA	Quinolinic acid/kynurenic acid ratio	QA and KA are the two end products of the metabolism of tryptophan	serum	disease severity	The QA/KA ratio positively correlates with EDSS (r=0.62, p<0.0001).	103 RR, 55 SP, 17 PP	[277]	[54, 55]
Biomarkers of Innate Immunity	sCD14<br>sCD163<br>sCD21<br>sCD27	soluble CD14<br>soluble CD163<br>soluble CD21<br>soluble CD27	Soluble cell surface receptors from microglia and macrophages, released by monocytes	CSF	intrathecal inflammation	May be markers of microglial activation. Proposed as markers to monitor disease activity and response to treatment.<br>Area under the ROC curve for intrathecal T-cell mediated inflammation: CD14: AUC=0.72; CD163: AUC=0.66; CD21: AUC=0.77; CD27: AUC=0.97.<br>The reviewer cites only CD14 and CD163 as emerging biomarkers [26].	386 CSF samples	[278]	[26]
